# Synthesis of Natural-Inspired Materials by Irradiation: Data Mining from the Perspective of Their Functional Properties in Wastewater Treatment

**DOI:** 10.3390/ma16072686

**Published:** 2023-03-28

**Authors:** Mirela Braşoveanu, Hassan Sabbaghi, Monica R. Nemţanu

**Affiliations:** 1 National Institute for Laser, Plasma and Radiation Physics, 409 Atomiştilor St., P.O. Box MG-36, 077125 Bucharest-Măgurele, Romania; 2 Department of Food Science and Technology, Faculty of Agriculture, Ferdowsi University of Mashhad, Mashhad 9177948978, Razavi Khorasan Province, Iran

**Keywords:** biopolymer, copolymerization, flocculant, functionality, feature selection, correlation, principal components, prediction, statistical techniques

## Abstract

The present study is focused on assessing the interrelation of variables involved in the synthesis of natural-inspired copolymers by electron beam grafting while taking the functionality of the resulting materials into account. In this respect, copolymers of starch-graft-polyacrylamide (St-*g*-PAM) were synthesized by irradiation, and their flocculation efficiency regarding the total suspended solids (*TSS*), chemical oxygen demand (*COD*), and fatty matters (*FM*) was tested in coagulation–flocculation experiments at laboratory scale on wastewater from the oil industry. Data mining involved approaches related to the association (correlation and dimensionality reduction with principal component analysis (PCA)), clustering by agglomerative hierarchical clustering (AHC), classifying by classification and regression tree (CART), and prediction (decision tree prediction, multiple linear regression (MLR), and principal component regression (PCR)) of treatments applied with the variation of the monomer concentration, irradiation dose, and dose rate. The relationship mining proved that the level of *COD* was significantly affected by the irradiation dose and monomer concentration, and *FM* was mainly affected by the dose rate (significance level = 0.05). *TSS* showed the highest negative correlation with the tested variables. Moreover, the consequences of MLR demonstrated an acceptable accuracy (mean absolute percentage error < 5%) for *COD* and *FM*; meanwhile, linear modeling together with the consequences of PCA in the structure of PCR could help to simplify and improve the prediction accuracy of equations.

## 1. Introduction

Natural materials are abundant, renewable, and biodegradable, making them attractive options for a variety of applications in different areas of modern life. An important sector of life today is ensuring the ecological balance of water for human consumption. Wastewater is usually generated from residual water that is discharged from industries, households, or different places and generally includes components that can be unsafe to human health, affecting the activities of different living things and finally resulting in environmental damage or at least the potential to cause serious pollution problems and the deterioration of the ecological balance [1]. As this issue is turning into a top concern, advantageous treatment needs to be carefully explored to understand the most environmentally friendly approaches to wastewater treatment. Thus, wastewater treatment aims to exclude hazardous components from it and reduce/eliminate toxic compounds [2].

Coagulation and flocculation are processes commonly used in the treatment of wastewater. The coagulation–flocculation procedure is simple to operate and design, cost-effective, and reliable with low energy consumption [3]. Coagulation can produce the removal of components larger than colloidal particles due to the entrapment of such particles in the flocs formed by coagulation [4], while flocculation refers to the procedure used to transport coagulated particles collectively so that they can mix to create larger, filterable, and settleable masses of particles called floc. Therefore, coagulants and flocculating agents are used in effluent wastewater treatment methods for solids removal, water clarification, lime softening, sludge thickening, mineral processing, solids dewatering, and sample processing for monitoring applications [5].

Polymeric materials with flocculating properties can aggregate particles into larger clusters (flocs). Thus, polymers can be used as a “coagulant aid” to enhance the overall performance of coagulants with the aid of building and constructing the bridges between particles resulting from coagulants’ activities, creating large, massive, and heavy clots and accelerating the sedimentation process [6]. Based on this, they are widely used in the processes of potable water and wastewater treatment [7,8,9], having the major advantage of decreasing coagulant consumption [10]. Natural polymers such as cellulose, starch, or chitosan can be used as the base materials of biopolymer-based flocculants in wastewater treatment due to their characteristics, concentration, molecular weight, nature, and chemical composition in water treatment efficiency [8,11,12]. Starch-based materials are often chosen for wastewater treatment as a coagulant or flocculant due to their renewable and less expensive raw materials, as well their availability and biodegradability in comparison with synthetic materials [13,14,15].

The synthesis of starch-based materials involves the modification of starch through different chemical, physical, and enzymatic methods, or dual combinations of these methods [16]. One of the most used methods for the synthesis of such materials as flocculating agents is the graft copolymerization of starch with vinyl monomers, which results in the production of copolymers with flocculating features. The radiation-induced synthesis of polymeric-based materials in general, and starch graft copolymers in particular, offers several advantages over other synthesis methods, including high efficiency and specificity, improved molecular control, and reduced environmental impact [17,18,19]. However, the specific parameters, such as the monomer concentration, irradiation dose, and irradiation dose rate, used in this kind of synthesis can affect the functional properties of the resulting copolymers and should be carefully controlled and optimized to ensure optimal performance.

It is well known that, nowadays, analysis, inter-correlation, modeling, and optimization methods are widely used in various fields such as materials science and engineering, manufacturing and production, chemical and biomedical engineering, and environmental science to improve the efficiency, quality, and performance of products and processes while reducing costs as well. In materials science, these methods help to identify and understand the relationships between input variables (i.e., processing parameters, raw material parameters) and the target variables (resulting material properties), which can be used to optimize the processing conditions for specific applications. Several types of statistical methods such as linear or nonlinear regressions, principal component analysis (PCA), partial least squares regression (PLS), response surface methodology (RSM), artificial neural networks (ANN), and support vector machines (SVM) have been reported in recent years to be used for the analysis, correlation, modeling, optimization, and data mining of experimental observations in various fields [20,21,22,23,24].

In our previous reports on the synthesis and optimization of the production process of starch-based copolymers [25,26,27,28], the optimization of this process was carried out concerning some of the physicochemical characteristics of the synthesized copolymers, such as the residual monomer concentration, monomer conversion coefficient, and intrinsic and apparent viscosities. In these cases, the implementation of different multicriteria optimization strategies was generally pursued mainly by unifying requirements for economic efficiency and ensuring low toxicity and high copolymer efficiency in the flocculation process. The optimization methodologies successfully involved regressions and neural network-based models, an overall robust desirability function, scanning and genetic algorithms, and graphical optimization.

However, the current work proposes another way to investigate the interrelation with data mining in the synthesis of starch-based copolymers by electron beam grafting—more precisely, from the perspective of the functionality of the resulting materials. This approach of the process of refining the performance of the radiation-synthesized starch-based copolymers to enhance their flocculating abilities by correlating input variables (i.e., mixture composition, processing parameters) with functional outputs (flocculation efficiencies) and further optimizing processing conditions has not been reported to date, according to our knowledge. This is also supported by Jiang and collaborators [11], who, in a recent review, stated that future studies related to biopolymer-based flocculants should be mainly focused on the optimization of modification processes to improve the flocculation performance of such materials and their multi-functionality.

Therefore, the main objective of the current study was to evaluate the relationships among the input processing parameters, namely, the monomer concentration, irradiation dose, and dose rate, for starch-graft acrylamide copolymer radiation synthesis by using data mining techniques to obtain desired flocculation abilities concerning the total suspended solids, chemical oxygen demand, and fatty matters of real wastewater. To achieve this goal, the following were pursued: (1) a dimensionality reduction using the Principal Component Analysis technique; (2) developing a classification model of observations based on the degree of correlation to principal components with the Agglomerative Hierarchical Clustering method; (3) creating a regression decision tree to evaluate the reduction in features, which could help in providing the best-related variables in optimizations, future research, and modeling to enhance accuracy; (4) finally, performing Multiple Linear Regression on the original data (original features) and then performing Principal Component Regression on the principal component outputs (reduced features) to compare the prediction power of reduced dimension data. This novel path of using data mining methods to refine the performance of radiation-synthesized materials by correlating input variables with functional outputs and optimizing processing conditions can minimize the number of experiments and save time. Additionally, data mining, which is also known as Knowledge Discovery of Data (KDD), can assist in predicting the potential effects in the application of new treatments, determining the strategies of the irradiation process, and improving or developing the decision-making systems.

## 2. Materials and Methods

### 2.1. Materials

Starch from potato (S4251; powder) was purchased from Sigma-Aldrich (St. Louis, MO, USA), and acrylamide (A17157; 98+%; white; crystalline) was purchased from Alfa Aesar (Karlsruhe, Germany). Other chemicals were of analytical grade and purchased from SC Chimreactiv SRL (Bucuresti, Romania). The materials used to prepare the copolymers and their characteristics are presented in Table 1. 

### 2.2. Radiation-Induced Synthesis of Copolymers 

The synthesis of copolymers was carried out according to the methodology described by Nemţanu et al. [19], with some slight modifications. Thus, starch samples (1.7% *w*/*v*) were prepared by gelatinizing powder starch in distilled water in a water bath at 85 °C with continuous magnetic stirring for 30 min. After cooling the starch samples to room temperature (23 ± 1 °C), acrylamide and sodium chloride were added with further stirring, obtaining homogeneous mixtures of potato starch:acrylamide (PS:AMD) with weight ratios of 1:6 and 1:12, respectively. The resulting mixtures were divided into two different batches depending on the PS:AMD ratio and marked accordingly: *batch 1* for PS:AMD = 1:6 and *batch 2* for PS:AMD = 1:12, respectively. Each batch contained nine samples, which were further subjected to electron beam irradiation with different input parameters in a static mode. Sample irradiation was performed with a linear accelerator of a mean energy of 6 MeV (ALIN-10, NILPRP, Bucharest-Măgurele, Romania) using different irradiation doses (*D* = 0.6–2.7 kGy) and dose rates ($\dot{D}$ = 0.7–1.9 kGy/min) at room temperature (23 ± 1 °C) and ambient pressure under air. The recipe and process variables were selected based on our previous investigations related to the grafting of starch in the radiation field for the synthesis of water-soluble copolymers [19,20,29]. 

For each batch, the marking of the PS-*g*-6AMD- and PS-*g*-12AMD-type samples was carried out following the increasing order of the irradiation dose: PS-*g*-6AMD_1…PS-*g*-6AMD_9 and PS-*g*-12AMD_1…PS-*g*-12AMD_9.

### 2.3. Flocculation Investigation

The copolymer functional parameters were evaluated according to standardized methods [30,31,32] in coagulation–flocculation experiments on wastewater collected from an oil processing plant. The coagulation–flocculation experiments were performed at the laboratory level, using classic inorganic coagulants (200 mg/L CaCO_3_ and 200 mg/L Al_2_(SO_4_)_3_) and a dosage of 2 mg/L of a 0.2% aqueous solution copolymer (flocculant). The quality parameters investigated in this study were pH, total suspended solids (*TSS*), chemical oxygen demand (*COD*), and fatty matters (*FM*). The flocculation efficiency (*FE%*) for each parameter was determined with Equation (1):

(1) $$FE\%=\frac{C_{0}-C}{C_{0}}\times 100$$

where *C*_0_ and *C* are the concentrations (in mg/L) of the investigated parameter before and after the tested water treatment.

### 2.4. Data Mining

The statistical analysis dedicated to the correlation of the variables, the dimensionality reduction, the classification of the treatments applied with the variation of the PS:AMD ratio, the irradiation dose, and the dose rate, as well as the linear modeling, was carried out based on the methods described further in this section. Table 2 briefly shows the coding of the treatments for the resulting copolymers involved in coagulation–flocculation tests.

#### 2.4.1. Correlation Matrix

The linear correlation between independent (PS:AMD ratio, *D*, and $\dot{D}$) and dependent (*TSS*, *COD*, and *FM*) variables was studied using both Pearson’s correlation coefficient and Spearman’s rank correlation coefficient with the software of IBM SPSS Statistics V22.0. 

Pearson’s correlation coefficient *r* was calculated using Equation (2):

(2) $$r=\frac{n\left(\sum xy\right)-\left(\sum x\right)\left(\sum y\right)}{\sqrt{\left[n\sum x^{2}-{\left(\sum x\right)}^{2}\right]\left[n\sum y^{2}-{\left(\sum y\right)}^{2}\right]}}$$

where *x* and *y* are the values of the *x*-variable and the *y*-variable, respectively, and *n* is the number of the pairs of values [33].

Spearman’s rank correlation coefficient *r_s_* was calculated using Equation (3):

(3) $$r_{s}=r_{R\left(x\right),R\left(y\right)}=1-\frac{6\sum d_{i}{}^{2}}{n\left(n^{2}-1\right)}$$

where $r_{R\left(x\right),R\left(y\right)}$ denotes the usual Pearson’s correlation coefficient, but applied to the rank *R* variables, *d_i_* = *R(x_i_)* − *R(y_i_)* is the difference between the two ranks *R* of each observation, and *n* is the number of observations [34].

#### 2.4.2. Bartlett’s Sphericity Test

Bartlett’s test of sphericity examined the hypothesis that the correlation matrix is an identity matrix, which would point out that variables are unrelated and therefore unsuitable for structure detection [35,36]. Equation (4) was indicated for the Chi-square (*χ*^2^) value, where *n* is the number of observations, *p* is the number of variables, and *R* is the correlation matrix. The *χ*^2^ test was then performed on “(*p*^2^ − *p*)/2” and “the total number of variable pairs minus one or ([*p* + (*p* − 1) + (*p* − 2)+…+(*p* − *p*)] − 1)” degrees of freedom (DF) based on Pearson’s correlation coefficient *r* and Spearman’s rank correlation coefficient *r_s_*, respectively. It was considered that the determinant of the correlation matrix will be equal to 1.0 only if all correlations are equal to 0; otherwise, the determinant will be less than 1. Furthermore, the test interpretation was: H_0_: There is no correlation significantly different from 0 between variables; H_a_: At least one of the correlations between the variables is significantly different from 0. Thus, if the computed *p*-value is lower than the significance level alpha = 0.05, then the null hypothesis H_0_ should be rejected and the alternative hypothesis H_a_ accepted [37]. The IBM SPSS Statistics V22.0 software was also used to perform Bartlett’s test.

(4) $$\chi^{2}=-\left(n-\frac{1}{6}\left(2p+5\right)\right){log}_{e}\left|R\right|$$

#### 2.4.3. Principal Component Analysis (PCA)

In order to reduce the dimensions of the study, principal component analysis was performed among the studied components by XLSTAT statistical software V21.5. To obtain the principal components, first, the data have been standardized using Equation (5) such that any point *x_i_* from a normal distribution can be converted to the standard normal distribution *Z*:

(5) $$z_{i}=\frac{x_{i}-x_{m}}{s_{i}}$$

where *Z_i_* is the standardized variable, and *x_m_* and *s_i_* are the mean and standard deviation of each variable, respectively [38].

Principal component analysis generally transforms the original dataset of *n* variables, which are correlated among themselves to various degrees, into a new dataset containing *n* number of uncorrelated principal components (PCs). The PCs are linear functions or linear features (F) of the original variables in such a way that the sums of the variances are equal for both the original and new variables. The PCs are sequenced from highest to lowest variance. The first PC explains the largest amount of variance in the data. The subsequent highest variance is explained by the second PC, and so on for all *n* PCs. The values of all PCs can be obtained by the same equation as Equations (6) and (7) for PC1 (F1) and PC2 (F2), respectively, where *x_1_*, *x_2_*, … *x_n_* are the original variables in the dataset and *a_jj_* are the eigenvectors. Although the numbers of PCs and the original variables are equal, normally, most of the variance in the dataset can be defined by the first few PCs that can be used to represent the original observations. Finally, PCA helps in decreasing the dimensionality of the original dataset [39,40].

(6) $$PC1=F1=a_{11}x_{1}+a_{12}x_{2}+...+a_{1n}x_{n}=\sum_{j=1}^{n}a_{1j}x_{j}$$

(7) $$PC2=F2=a_{21}x_{1}+a_{22}x_{2}+...+a_{2n}x_{n}=\sum_{j=1}^{n}a_{2j}x_{j}$$

The eigenvalues are the variances of the PCs, and the coefficients *a_jj_* are the eigenvectors extracted from the covariance or correlation matrix of the dataset. The eigenvalues of the data matrix can be calculated using Equation (8), where *C* is the correlation/covariance matrix, *λ* is the eigenvalue associated with the eigenvector, and *I* is the identity matrix [41,42].

(8) $$\left|C-\lambda I\right|=0$$

The PC coefficients, or the weights of the variables in the PC, are then calculated by using Equation (9).

(9) $$\left|C-\lambda I\right|a_{jj}=0$$

In our study, a correlation matrix of the variables was used to gain eigenvalues and eigenvectors. The eigenvectors multiplied through the square root of the eigenvalues produce an *n* × *n* matrix of coefficients, which are referred to as variable loadings. The importance of each original variable to a specific PC is represented by means of these loadings. Furthermore, the sum of the products of the variable loadings and the values of the original variables produces a new set of data values, which are known as component scores or factor scores. These scores can be used in multiple linear equations as new variables to predict outputs as future variables [42].

#### 2.4.4. Agglomerative Hierarchical Clustering (AHC)

The classification of the tested treatments by varying the PS:AMD ratio (1:6 and 1:12, respectively), irradiation dose (*D* = 0.6–2.7 kGy), and dose rate ($\dot{D}$ = 0.7–1.9 kGy/min) was performed using agglomerative hierarchical clustering in a bottom-up approach using the software of MATLAB 2022a (R2022a), based totally on the squared cosine values from the PCA. Thus, the treatments were divided into several clusters such that the data points from the same cluster were more similar (more comparable) and the data points from different clusters were dissimilar. In general, the basis of many measures of similarity and dissimilarity is Euclidean distance. The distance between the vectors X and Y is described as the square root of the sum of the squared differences between the corresponding elements of the two vectors. Ward’s method was applied as a general AHC procedure, where the criterion for choosing the pair of clusters to be merged at each step is based on the optimum value of an objective function [43].

#### 2.4.5. Decision Tree Prediction

The regression tree algorithm as the classification and regression tree (CART) was used to find one learning model that results in good predictions for the new data of *TSS*, *COD*, and *FM* and to discover the best probability conditions in simultaneous data mining that ensure the fitting of the dependent investigated within limits allowed by the regulation (*TSS* ≥ 70%, *COD* ≥ 85%, and *FM* ≥ 85%). The decision tree was made with the CHAID method using the software of XLSTAT statistical V21.5 under the following conditions [44]: significance level of 5%, Split threshold of 5%, and authorized redivision: Bonferroni correction/Merge threshold of 5%. Finally, optimization rules were obtained for inputs (PS:AMD ratio, *D*, and $\dot{D}$) and outputs (*TSS*, *COD*, and *FM*).

#### 2.4.6. Multiple Linear Regression (MLR)

Multiple linear regression analysis attempted to model the relationship between two or more independent variables and a dependent variable with XLSTAT statistical software V21.5 by fitting a linear equation to the observed data. The conventional equation of an MLR model can be expressed as Equation (10) [42,45]:

(10) $$Y=a_{0}+a_{1}x_{1}+a_{2}x_{2}+\ldots +a_{n}x_{n}$$

where *Y* is the dependent variable (*TSS*, *COD*, or *FM*), *a_i_* (*i* = 0, … n) are the parameters generally estimated by the least squares method, and *x_i_* (*i* = 0, … n) are the independent variables (PS:AMD ratio, *D*, and $\dot{D}$).

#### 2.4.7. Principal Component Regression (PCR)

In principal component regression, MLR and PCA are usually combined to set up a relation between the dependent variable *Y* and the selected PCs (Fs) of the input variables. Thus, the principal component scores (factor scores) obtained from the PCA were taken as the independent variable in the multiple linear regression equation to operate the PCR analysis with XLSTAT statistical software V21.5. The general function of a PCR model is according to Equation (11) [42,45].

(11) $$Y=a_{0}+a_{1}F_{1}+a_{2}F_{2}+\ldots +a_{n}F_{n}$$

#### 2.4.8. Models’ Evaluation

The performances of the developed MLR and PCR models were measured and compared using the mean absolute percentage error (MAPE) according to Equation (12):

(12) $$MAPE=\frac{1}{n}\sum_{i=1}^{n}\left|\frac{P-O}{O}\right|\times 100$$

where *O* indicates the observed data, *P* shows the predicted value of the model, and *n* is the number of observations [46].

## 3. Results

### 3.1. Flocculation Performances

The synthesized copolymers had generally good performances in the coagulation–flocculation process. The pH value of raw water decreased from 8.6 to 7.7 by adding inorganic coagulants. No significant alteration of the pH value was recorded after flocculant addition, the tested water having a pH value of 7.7 ± 0.2. Therefore, the water treated by the coagulation–flocculation process fell within the limits allowed by the regulation, and the dosage of copolymers used in this study did not affect the pH resulting from the coagulation process of the residual water.

The copolymer presence increased the *TSS* yield by up to approximately 18% in addition to the efficiency of the inorganic coagulants. In general, copolymer efficiencies of more than 10%, which practically brought the *TSS* within the limits allowed by the regulation, were observed for both batches at irradiation doses of 0.6–1.4 kGy for PS-*g*-6AMD samples and 0.6–0.8 kGy for PS-*g*-12AMD samples, with dose rate ranges of 0.7–1.2 kGy/min and 0.9–1.2 kGy/min, respectively. These findings show that the increase in the AMD concentration contributed to the reduction in the irradiation dose range with the narrowing of the dose rate range.

The *COD* yield was only slightly increased up to about 5% by adding copolymers to inorganic coagulants in the water treatment. However, the classic treatment with coagulants selected for this study generally managed, by itself, to ensure the maximum level allowed by the regulation. Therefore, the application of the synthesized copolymers made an additional contribution to the decrease in the *COD* level in the treated water. Efficiencies of 4–5% after the application of the coagulation process were obtained for the PS-*g*-6AMD sample exposed to 0.9 kGy with 2.1 kGy/min and for the PS-*g*-12AMD samples irradiated with doses of 0.9–1.2 kGy at a dose rate of 0.7–1.0 kGy/min. This result indicates that the range of irradiation doses was extended along with the dramatic reduction in the dose rate by increasing the starch-to-monomer ratio.

The *FM* yield was also increased by using the synthesized copolymers in water treatment after the coagulation process. An efficiency of over 15% of the added flocculant ensured that the water fell within the maximum level allowed according to the regulation. Thus, efficiencies > 15% were observed for PS-*g*-6AMD samples irradiated at 0.9–2.7 kGy with 1.4–1.9 kGy/min and for PS-*g*-12AMD samples irradiated at 1.2–1.4 kGy with 0.7–0.9 kGy/min, respectively. Based on these results, it was understood that, for a good copolymer efficiency for *FM*, the range of irradiation doses required for copolymer synthesis, regardless of the AMD concentration, was higher than that for the other quality parameters. At the same time, the dose rate decreased significantly with an increase in the starch-to-monomer ratio.

This investigation showed that the copolymers synthesized in this work had flocculation capabilities and were effective in reducing the quality parameters (*TSS*, *COD*, and *FM*) of the wastewater collected from an oil factory. Copolymers with a lower acrylamide content (PS-*g*-6AMD) showed better results for *TSS* and *FM* parameters compared to those with a high acrylamide content (PS-*g*-12AMD), which instead showed better results for *COD*. However, it should be noted that the copolymers of *batch 2* (with a high AMD content) with a satisfactory efficiency in reducing all quality parameters required lower irradiation parameters compared to efficient copolymers from *batch 1*, namely, irradiation doses of 0.6–1.4 kGy with dose rates of 0.7–1.2 kGy. The obtained result is consistent with previous studies [47], which reported that samples with a higher AMD content require lower irradiation doses, thus leading to the formation of longer grafted polyacrylamide chains that can ensure better efficiency in reducing wastewater quality parameters as a result of a higher molecular weight and intrinsic viscosity.

### 3.2. Correlation Investigation

The correlation matrices for the tested variables, based primarily on Pearson’s *r* and Spearman’s rank *r_s_* correlation coefficients, are given in Table 3 and Table 4, respectively. Generally, only very weak to moderate correlations were found between the tested treatments (processing parameters) and the output variables (functional properties). However, the highest significant correlations based totally on the Pearson’s coefficient *r* were found between (PS:AMD ratio and *COD*) and (*D* and *COD*), with values of 0.541 and 0.515, respectively (Table 3). These results indicate that *COD* is positively correlated with both the monomer concentration and irradiation dose, but without a significant influence of the dose rate. Conversely, a correlation between the PS:AMD ratio and *COD* was not observed according to the Spearman’s rank correlation coefficient (Table 4), while it was found that *r*_s_ > r for the correlation of *COD* with *D*. 

On the other hand, the lowest correlation (negative correlation) was found based on both Pearson’s and Spearman’s rank correlation coefficients for (*COD* and *TSS*). This observation shows that these two functionalities vary inversely proportionally depending on the number and nature of the inorganic solids present, the nature of organic solids, and the quantity of dissolved organic matter. Therefore, a constant low variance correlation between *COD* and *TSS* could not be observed. Moreover, *COD* and *TSS* are totally different parameters, and thus, no positive correlation between them is expected [48].

To select the appropriate correlation coefficient for the subsequent operation of the PCA test, we ought to pay attention to the consequences of Bartlett’s sphericity test, which are displayed in Table 5 for our study. The *p*-value indicates that the risk of rejecting hypothesis H_0_ while it is true (type I error) [49] by using Spearman’s rank correlation coefficient is less than 0.82%, which will provide a more dependable and reliable result compared to the Pearson correlation coefficient (type I error < 1.17%). Therefore, Spearman’s rank correlation coefficient was used in our study for PCA.

### 3.3. Dimensionality Reduction Study

**Figure 1:**
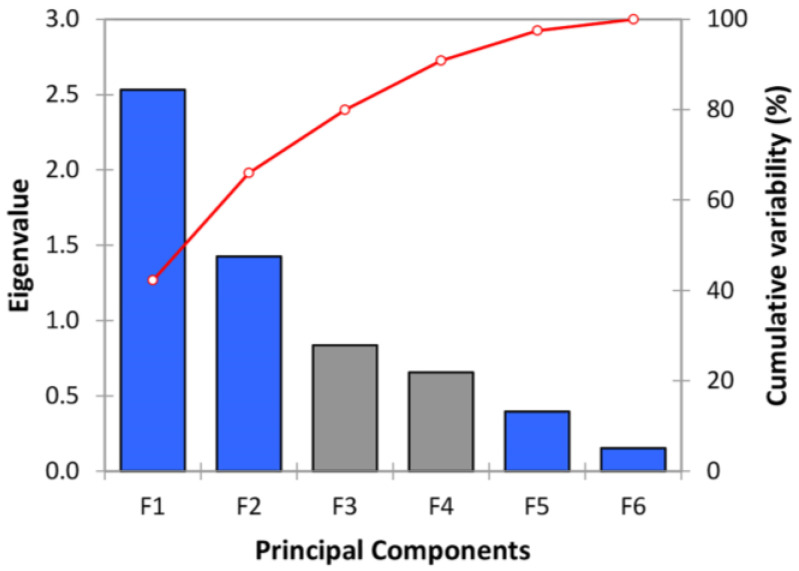
Scree plot for explained eigenvalues and cumulative variability (%) from PCA analysis.

A scree plot in accordance with Figure 1 indicates the eigenvalues on the y-axis and the number of factors on the x-axis. Eigenvalues represent and characterize the magnitude or importance of the eigenvectors. The point where the slope of the curve certainly levels off (the “elbow”) suggests the number of factors to be generated with the analysis. Thus, in our analysis, the cumulative variability (red curve in Figure 1) was equal to 79.931% (~80%) and 90.866% (~91%) after the third (F3) and fourth (F4) principal components (PCs), respectively. Therefore, the number of three or, strictly speaking, four factors seems appropriate for reducing the dimensions, considering that the optimal minimum cumulative variability to decide on the number of factors is equal to 80% [50].

In the next step, the matrix of eigenvectors (*a_jj_*) was generated (Table 6). The eigenvalue indicates the quantity of variability in the direction of its corresponding eigenvector. Therefore, the eigenvector with the largest eigenvalue is the direction with the most variability, and this eigenvector is the first principal component (F1). 

Furthermore, the matrix of factor loadings was provided according to Table 7. The weights are the correlation between the standardized scores of the variables and the principal components, also recognized as factor loadings. The factor loading is the level of correlation existing between each variable and the corresponding factor [51]. A factor loading of greater than 0.30 commonly suggests a moderate correlation between the variable and the factor, while a higher factor loading represents that the factor extracts sufficient variance from that variable [52]. Thus, it was observed that the factor loading values for all variables, except *TSS*, indicate an increase in their contribution, especially for *D* and *COD*, to the increase in F1. It should also be mentioned that although the factor loading values for some variables, such as $\dot{D}$ and *FM*, showed contributions to the factor increase in three of the four factors that cover ~90% of the variability, the greater contribution was observed within a single factor (principal component), namely, F4 and F3, respectively.

Additionally, a negative loading simply means that a certain attribute (variable) is a lack of correlation as a variable associated with the given principal component [53]. For example, such variables with higher factor loading values were *TSS* in the case of F1 and PS:AMD for F2.

**Figure 2:**
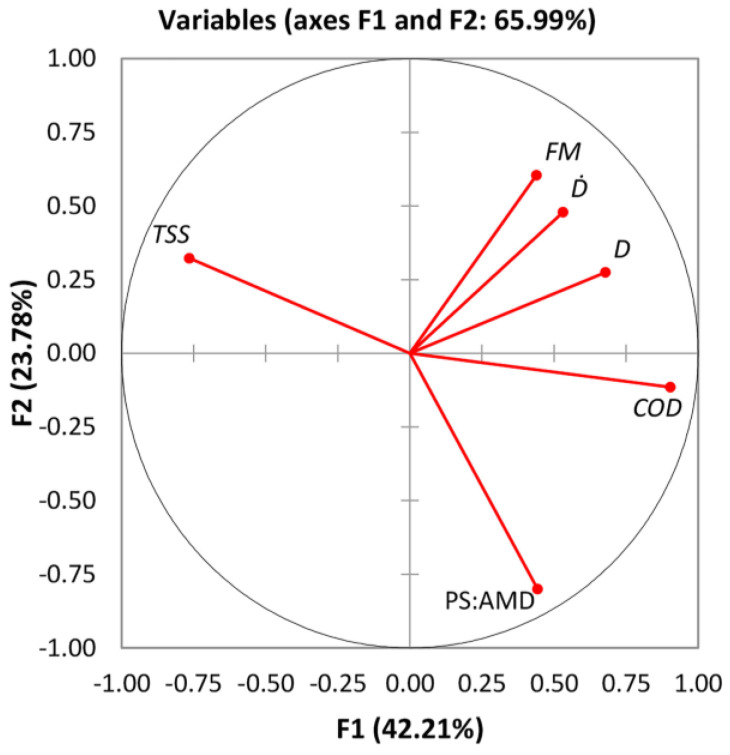
Correlations circle between study variables and factors.

The correlation circle between the features of the original dataset and the first two principal components (F1 and F2 ~66% of the cumulative variability) is displayed in Figure 2. It can be easily observed that *FM* and *COD* are the variables that are positively correlated with *D* and $\dot{D}$, all being grouped. Instead, *TSS* correlates negatively with all processing variables, being located on the opposite facet of the plot origin (opposed quadrant). Moreover, it can be observed that *COD* shows a higher positive correlation with *D* and PS:AMD, as indicated by the small angle formed with these variables. These consequences are also consistent with the results in Table 4.

The percentage contribution of each studied variable to each principal component is given in Table 8. This is basically a scaled version of the squared correlation between variables and component axes (or cosine, from a geometrical point of view), which is generally used to investigate the quality of the illustration of the variables of the principal component.

The squared cosines of the study variables for the quality of representation on the factor map are shown in Table 9. As can be observed, for each variable, the largest of the squared cosines up to the fourth factor was obtained as follows: **F1**: *D*, *TSS*, and *COD*; **F2**: PS:AMD ratio; **F3**: *FM*, and **F4**: $\dot{D}$, which represents the correlation of these variables with the respective principal component (or axis).

**Figure 3:**
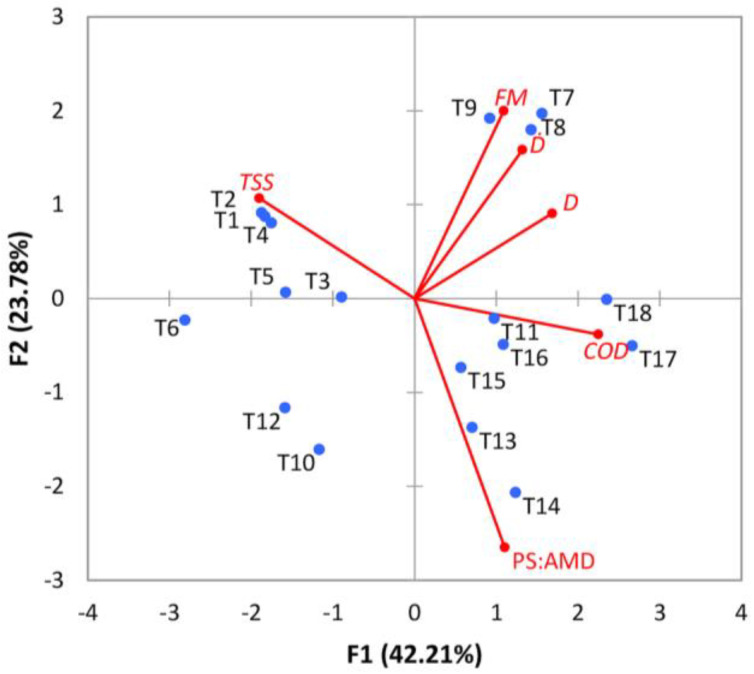
Factor scores in PCA Biplot (treatments T1…T18).

The PCA biplot for the treatments tested in our study is shown in Figure 3. The plot shows the treatments (T1…T18) as points primarily based on factor scores and the original variables (PS:AMD ratio, *D*, *Ḋ*, and *TSS*, *COD*, *FM*) as vectors in the plane formed through the first two principal components (F1 and F2). It was thus noticed that the treatments with higher *TSS*, *COD,* or *FM* efficiencies are displayed under the influence of their respective vectors. Moreover, the treatments that led to higher *TSS* efficiencies are located on the left face of the coordinates (T1…T6, T10, T12), while the treatments that led to high *COD* efficiencies are marked on the right side of the coordinates (T7…T9, T11, T13…T18).

Table 10 shows the factor scores for all tested treatments, pointing out their placement in the coordinate system made of the desired principal component. For example, in the case of F1 and F2, the factor score values in Table 10 are consistent with the PCA Biplot (Figure 3).

Table 11 shows the squared cosines of the observations (T1…T18) for output variables versus principal components. The excessive squared cosine suggests a proper representation of the variable on the principal component as follows: **F1**: T1, T2, T4…T6, T12, T17, T18; **F2**: T7…T10, T13, T14; **F3**: T11; **F4**: T16; **F5**: T3, T15. These results were further used for the treatment classification by AHC clustering.

### 3.4. Treatment Classification

**Figure 4:**
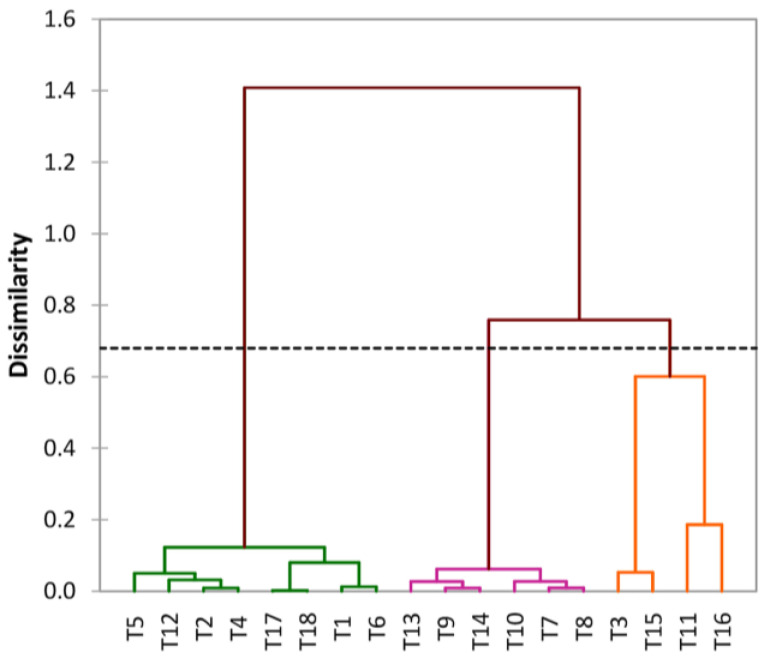
AHC Dendrogram of treatments.

The dendrogram generated based totally on PCA squared cosines (Figure 4) indicates the possibility of grouping all investigated treatments into three major clusters at a cut-off of about 0.680. Cluster 1 included eight treatments; cluster 2 included four treatments; and cluster 3 consisted of six treatments (Table 12). It has also been observed that cluster 1 mainly included treatments corresponding to *batch 1*, while cluster 2 grouped mainly treatments corresponding to *batch 2*, and treatments corresponding to both batches were equally found in cluster 3.

**Figure 5:**
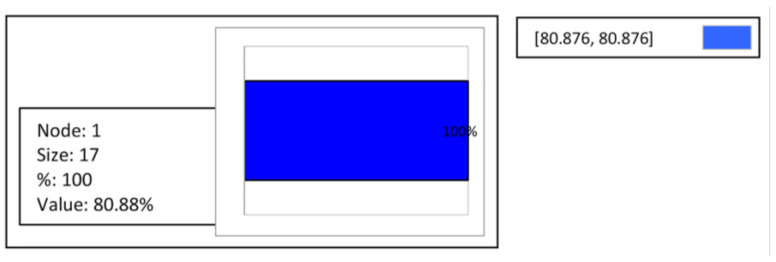
Regression tree: decision making for *TSS*.

Furthermore, for the regression tree achievement, the investigated functional variables within the limits allowed by the regulation (*TSS* ≥ 70%, *COD* ≥ 85%, and *FM* ≥ 85%) were considered to ensure the best possible fitting. Figure 5 shows the consequences of the regression tree for *TSS*, indicating that the predicted value was equal to 80.88%, including 100% of cases with a node size of 17, which means that *TSS* ≥ 70% under the test conditions. 

**Figure 6:**
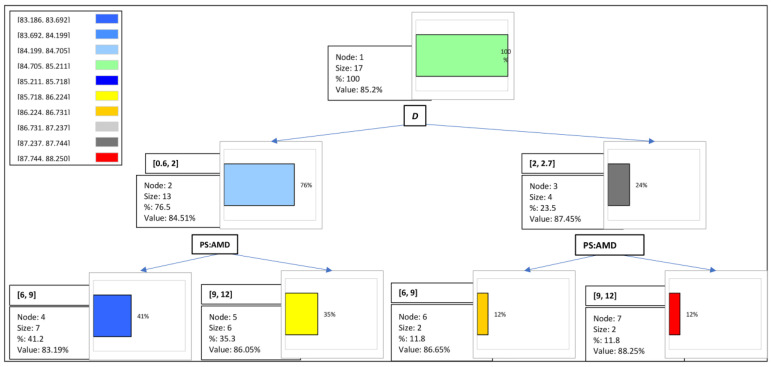
Regression tree: decision making for *COD*.

The results of the regression tree for *COD* are presented in Figure 6, and the rules of its decision tree are additionally shown in Table 13. As observed previously (Figure 2), the *COD* value was affected by both the irradiation dose and monomer concentration. Under test conditions, the predicted value for *COD* was 85.2%. It has been found, however, that the impact of *D* on the amount of *COD* has priority over PS:AMD, and if *D* is in [2, 2.7], then *COD* = 87.450%. The highest value of *COD* equal to 88.25% is expected if the value of PS:AMD is between 1:9 and 1:12 (PS:AMD = [9, 12]) and, at the same time, *D* is between 2 and 2.7 kGy (*D* = [2, 2.7]) so that, subsequently, *COD* ≥ 85% under the conditions described.

**Figure 7:**
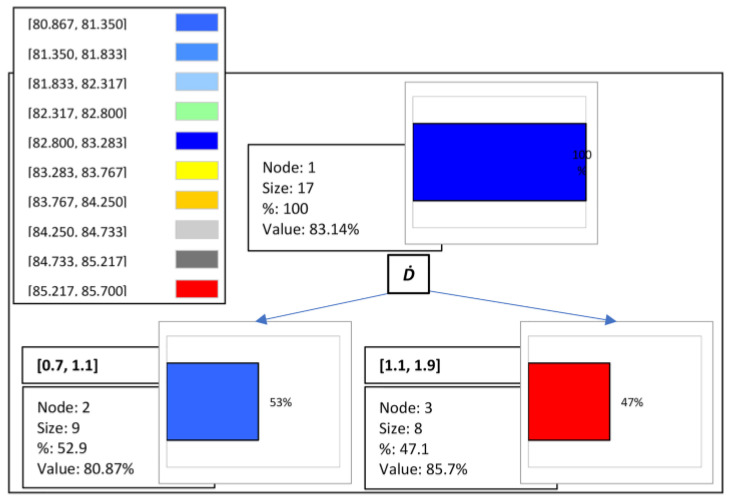
Regression tree: decision making for *FM*.

The regression tree results for *FM* are displayed in Figure 7, while the rules of the decision tree are shown in Table 14. As was shown, the value of *FM* was mainly affected by the change in $\dot{D}$. Therefore, the analysis suggested that, if $\dot{D}$ in [1.1, 1.9], then *FM* = 85.7% in 47.1% of cases, fulfilling *FM* ≥ 85% under these conditions.

### 3.5. Linear Modeling

**Figure 8:**
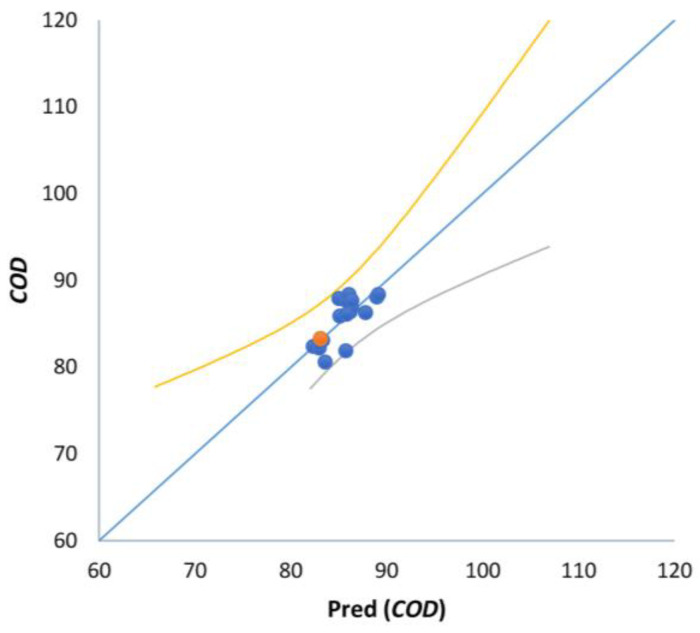
MLR training set in prediction of study variables versus empirical data (Case = *COD*).

The regression models based totally on MLR and PCR are provided in Table 15. The equations primarily based on the main variables (PS:AMD, *D*, and $\dot{D}$) confirmed the highest accuracy in *COD* and *FM* prediction, with MAPE equal to 1.412% and 4.167%, respectively. For example, Figure 8 shows the learning set for MLR in *COD* prediction. In the case of *TSS*, even though the MAPE was larger and equal to 8.842%, it nevertheless confirmed acceptable accuracy. 

Regression equations for PCR based on two, three, and four components are also shown (Table 15). The accuracy of PCR was better than that of MLR in all cases. In general, the use of the equation with two principal components (F1 and F2) can easily predict *TSS, COD*, and *FM* variables, with MAPE equal to 5.521, 0.991, and 2.710, respectively. The accuracy of PCR prediction always improved as the number of principal components increased, and these changes were much greater for *FM*, especially with the addition of F3, which could be because the squared cosines for *FM* were higher in the third principal component (Table 9). Therefore, PCR was successful in simplifying the prerequisites for predicting variables (*TSS*, *COD*, and *FM*) based on principal components (MAPE ≤ 5%).

## 4. Conclusions

The main findings of this work are summarized as follows:

1. The starch-based copolymers synthesized in this work using different monomer concentrations, irradiation doses, and dose rates proved to have effective flocculation properties by reducing the quality parameters (*TSS*, *COD*, and *FM*) of the wastewater of an oil factory.
2. The correlation between the input processing variables such as the PS:AMD ratio, *D*, and $\dot{D}$ and the flocculation efficiency of the synthesized copolymers regarding *TSS*, *COD*, and *FM* showed that *TSS* has an excessively negative correlation with other variables, *COD* is positively correlated with both the monomer concentration and irradiation dose, and *FM* demonstrated a moderately positive correlation with the dose rate.
3. The principal component analysis was able to correctly classify the correlation between the input processing variables and the target variables (copolymer functionalities) and determined the clustering of the treatments that had similar behavior as the principal components. High cumulative variability of ~80% and even ~91% could be explained after F3 and F4 PCs, respectively, with a majority contribution (~66%) of the first two PCs. All investigated treatments were segregated into three major clusters, of which cluster 1 included the largest number of treatments.
4. The analysis for meeting the allowed regulatory limits for the functional variables studied (*TSS* ≥ 70%, *COD* ≥ 85%, and *FM* ≥ 85%) of the copolymers synthesized in this work revealed that (i) *TSS* always had the desired level within the range of input processing variables; (ii) *COD* was influenced by the monomer concentration, but mostly by the irradiation dose, so the result was that an optimal *COD* value of 88.3% could be expected for a PS:AMD between 1:9 and 1:12 and an irradiation dose range of 2–2.7 kGy; (iii) *FM* was mainly affected by the dose rate, which, for the interval 1.1–1.9 kGy/min, could favor obtaining permissive conditions at 85.7%.
5. The consequences of linear modeling confirmed an acceptable accuracy for *COD* and *FM*, and the linear modeling along with the consequences of PCA in the structure of PCR could assist in simplifying the prediction equations.

Therefore, the functional efficiency of the starch-based flocculants synthesized by radiation-induced copolymerization depends on the processing parameters, which include both material parameters, such as the monomer concentration, and irradiation parameters, namely, the irradiation dose and dose rate. Using data mining methods related to association, clustering, classification, and prediction can considerably reduce the volume of experiments and save time regarding the appropriate parameter selection while also providing a major contribution to the design of machine learning algorithms, which can give substantial assistance, especially in industrial design and artificial intelligence, in the field of the synthesis of new natural-inspired materials involving radiation-based methods.

## Figures and Tables

**Table 1:** Substances used for radiation-induced graft copolymer synthesis.

Raw Material	Chemical Formula	Chemical Properties
Potato Starch (PS)	(C_6_H_10_O_5_)_n_	pH-test: 7.3 (2% suspension) Loss on drying: 18% Residue on ignition: 0.3%
Acrylamide (AMD)	CH_2_=CHCONH_2_ or C_3_H_5_NO	Molecular weight: 71.08 g/mol Density: 1.322 g/cm^3^ Boiling point: 125 °C/25 mm Melting point: 82–85 °C Flash point: 138 °C
Sodium chloride	NaCl	Molecular weight: 58.44 g/mol Density: 2.165 g/cm^3^ Boiling point: 1413 °C Melting point: 801 °C

**Table 2:** Treatment marking details.

Treatment Code	*Batch 1*	Treatment Code	*Batch 2*
T1	PS-*g*-6AMD_1	T10	PS-*g*-12AMD_1
T2	PS-*g*-6AMD_2	T11	PS-*g*-12AMD_2
T3	PS-*g*-6AMD_3	T12	PS-*g*-12AMD_3
T4	PS-*g*-6AMD_4	T13	PS-g-12AMD_4
T5	PS-*g*-6AMD_5	T14	PS-g-12AMD_5
T6	PS-*g*-6AMD_6	T15	PS-*g*-12AMD_6
T7	PS-*g*-6AMD_7	T16	PS-*g*-12AMD_7
T8	PS-*g*-6AMD_8	T17	PS-*g*-12AMD_8
T9	PS-*g*-6AMD_9	T18	PS-*g*-12AMD_9

**Table 3:** Correlation matrix (Pearson = *r*) of the studied variables.

Variable	PS:AMD	*D*	$\dot{D}$	*TSS*	*COD*	*FM*
PS:AMD	**1**	0.000	0.000	−0.435	**0.541**	−0.130
*D*	0.000	**1**	0.416	−0.312	**0.515**	0.249
$\dot{D}$	0.000	0.416	**1**	−0.208	0.385	0.300
*TSS*	−0.435	−0.312	−0.208	**1**	**−0.608**	−0.275
*COD*	**0.541**	**0.515**	0.385	**−0.608**	**1**	0.439
*FM*	−0.130	0.249	0.300	−0.275	0.439	**1**

Values in bold are different from 0 with a significance level alpha = 0.05.

**Table 4:** Correlation matrix (Spearman = *r_s_*) of the investigated variables.

Variable	PS:AMD	*D*	$\dot{D}$	*TSS*	*COD*	*FM*
PS:AMD	**1**	0.000	0.000	−0.471	0.461	−0.225
*D*	0.000	**1**	0.395	−0.308	**0.581**	0.156
$\dot{D}$	0.000	0.395	**1**	−0.184	0.296	0.313
*TSS*	−0.471	−0.308	−0.184	**1**	**−0.617**	−0.228
*COD*	0.461	**0.581**	0.296	**−0.617**	**1**	0.360
*FM*	−0.225	0.156	0.313	−0.228	0.360	**1**

Values in bold are different from 0 with a significance level alpha = 0.05.

**Table 5:** Bartlett’s sphericity test results based on Pearson *r* and Spearman *r_s_* correlation coefficients.

	*r*	*r_s_*
*χ*^2^ = Chi-square (Observed value)	30.064	38.273
*χ*^2^ = Chi-square (Critical value)	24.996	31.410
DF	15	20
*p*-value	0.012	0.008
Alpha	0.05	0.05
Risk to reject H_0_ while it is true (type I error)	<1.17%	<0.82%

**Table 6:** Eigenvectors matrix between the study variables and principal components.

Variable	F1	F2	F3	F4	F5	F6
PS:AMD	0.278	−0.670	0.010	0.334	0.383	−0.465
*D*	0.426	0.230	−0.582	−0.482	−0.006	−0.441
$\dot{D}$	0.334	0.401	−0.323	0.778	−0.084	0.106
*TSS*	−0.481	0.270	−0.278	0.038	0.785	−0.009
*COD*	0.568	−0.096	0.050	−0.219	0.417	0.666
*FM*	0.276	0.506	0.691	−0.017	0.236	−0.367

**Table 7:** Factor loadings based on PCA results.

Variable	F1	F2	F3	F4	F5	F6
PS:AMD	0.443	**−0.800**	0.009	0.271	0.241	−0.181
*D*	**0.678**	0.274	−0.532	−0.391	−0.004	−0.172
$\dot{D}$	0.531	0.479	−0.295	**0.630**	−0.053	0.041
*TSS*	**−0.765**	0.323	−0.254	0.030	0.494	−0.004
*COD*	**0.904**	−0.115	0.045	−0.177	0.262	0.260
*FM*	0.439	0.604	**0.632**	−0.014	0.149	−0.143

**Table 8:** Contribution of the studied variables (%) to each principal component.

Variable	F1	F2	F3	F4	F5	F6
PS:AMD	7.740	44.840	0.010	11.165	14.664	21.581
*D*	18.140	5.276	33.832	23.280	0.004	19.467
$\dot{D}$	11.132	16.051	10.407	60.584	0.702	1.123
*TSS*	23.127	7.306	7.738	0.141	61.679	0.009
*COD*	32.257	0.929	0.246	4.801	17.381	44.386
*FM*	7.604	25.598	47.766	0.028	5.569	13.434

**Table 9:** Squared cosines of the studied variables for the quality of representation on the factors map.

Variable	F1	F2	F3	F4	F5	F6
PS:AMD	0.196	**0.640**	0.000	0.073	0.058	0.033
*D*	**0.459**	0.075	0.283	0.153	0.000	0.030
$\dot{D}$	0.282	0.229	0.087	**0.398**	0.003	0.002
*TSS*	**0.586**	0.104	0.065	0.001	0.244	0.000
*COD*	**0.817**	0.013	0.002	0.032	0.069	0.067
*FM*	0.193	0.365	**0.400**	0.000	0.022	0.020

Values in bold correspond for each variable to the factor for which the squared cosine is the largest.

**Table 10:** Factor scores of the observations (T1…T18) for *TSS*, *COD*, and *FM* versus PCs (F1…F6).

Observations	F1	F2	F3	F4	F5	F6
T1	−1.832	0.875	1.128	0.051	0.029	−0.120
T2	−1.872	0.914	−0.188	0.511	0.438	0.440
T3	−0.892	0.017	0.659	0.011	−1.890	0.059
T4	−1.751	0.806	0.584	−0.803	0.300	−0.375
T5	−1.576	0.067	−0.228	−0.971	−0.463	0.027
T6	−2.809	−0.227	−1.822	−0.277	−0.642	0.028
T7	1.561	1.971	0.474	1.071	−0.174	0.883
T8	1.425	1.799	−0.269	−0.673	−0.314	−0.337
T9	0.920	1.922	−0.952	−0.734	0.589	−0.130
T10	−1.167	−1.608	−0.286	0.462	1.015	0.629
T11	0.972	−0.209	1.863	0.818	0.247	−0.436
T12	−1.585	−1.161	−0.425	0.794	0.224	−0.682
T13	0.703	−1.373	1.007	−0.619	0.333	0.100
T14	1.235	−2.063	0.530	−0.841	−0.579	0.491
T15	0.569	−0.732	0.506	−0.302	1.117	−0.042
T16	1.087	−0.487	−1.181	1.918	−0.185	0.401
T17	2.662	−0.504	−0.627	−0.179	−0.523	−0.407
T18	2.351	−0.007	−0.774	−0.238	0.477	−0.529

**Table 11:** Squared cosines of the observations (T1…T18) for *TSS*, *COD*, and *FM* versus PCs (F1…F6).

Observations	F1	F2	F3	F4	F5	F6
T1	**0.620**	0.141	0.235	0.000	0.000	0.003
T2	**0.698**	0.166	0.007	0.052	0.038	0.038
T3	0.165	0.000	0.090	0.000	**0.743**	0.001
T4	**0.622**	0.132	0.069	0.131	0.018	0.029
T5	**0.672**	0.001	0.014	0.255	0.058	0.000
T6	**0.672**	0.004	0.282	0.007	0.035	0.000
T7	0.286	**0.457**	0.026	0.135	0.004	0.092
T8	0.338	**0.539**	0.012	0.075	0.016	0.019
T9	0.133	**0.582**	0.143	0.085	0.055	0.003
T10	0.240	**0.456**	0.014	0.038	0.182	0.070
T11	0.176	0.008	**0.645**	0.125	0.011	0.035
T12	**0.484**	0.260	0.035	0.122	0.010	0.090
T13	0.127	**0.484**	0.260	0.098	0.028	0.003
T14	0.208	**0.580**	0.038	0.096	0.046	0.033
T15	0.132	0.218	0.104	0.037	**0.508**	0.001
T16	0.177	0.035	0.208	**0.550**	0.005	0.024
T17	**0.864**	0.031	0.048	0.004	0.033	0.020
T18	**0.826**	0.000	0.089	0.008	0.034	0.042

Values in bold correspond for each observation to the factor for which the squared cosine is the largest.

**Table 12:** AHC analysis results through class.

Class	1	2	3
Objects	8	4	6
Sum of weights	8	4	6
Within-class variance	0.044	0.280	0.027
Minimum distance to centroid	0.098	0.325	0.080
Average distance to centroid	0.189	0.452	0.145
Maximum distance to centroid	0.279	0.497	0.209
	T1	T3	T7
	T2	T11	T8
	T4	T15	T9
	T5	T16	T10
	T6		T13
	T12		T14
	T17		
	T18		

**Table 13:** Rules in decision tree for *COD*.

Node	Pred (*COD*%)	Frequency	Rules
Node1	85.200	17	-
Node2	84.508	13	If *D* in [0.6, 2], then *COD* = 84.508 in 76.5% of cases
Node3	87.450	4	If *D* in [2, 2.7], then *COD* = 87.450 in 23.5% of cases
Node4	83.186	7	If PS:AMD in [6, 9] and *D* in [0.6, 2], then *COD* = 83.186 in 41.2% of cases
Node5	86.050	6	If PS:AMD in [9, 12] and *D* in [0.6, 2], then *COD* = 86.050 in 35.3% of cases
Node6	86.650	2	If PS:AMD in [6, 9] and *D* in [2, 2.7], then *COD* = 86.650 in 11.8% of cases
Node7	88.250	2	If PS:AMD in [9, 12] and *D* in [2, 2.7], then *COD* = 88.250 in 11.8% of cases

**Table 14:** Rules in decision tree for *FM*.

Node	Pred (*FM*%)	Frequency	Rules
Node1	83.141	17	-
Node2	80.867	9	If $\dot{D}$ in [0.7, 1.1], then *FM* = 80.867 in 52.9% of cases
Node3	85.700	8	If $\dot{D}$ in [1.1, 1.9], then *FM* = 85.700 in 47.1% of cases

**Table 15:** Comparison between MLR and PCR models’ accuracy in the prediction of *TSS*, *COD*, and *FM*.

Regression	Models	MAPE%
MLR	*TSS* = 101.991 − (1.411 × PS:AMD) − (3.787 × *D*) − (2.494 × $\dot{D}$)	8.842
	*COD* = 77.320 + (0.461 × PS:AMD) + (1.569 × *D*) + (1.427 × $\dot{D}$)	1.412
	*FM* = 80.297 − (0.199 × PS:AMD) + (0.993 × *D*) + (2.961 × $\dot{D}$)	4.167
PCR	*TSS* = 81.022 − (4.580 × F1) + (2.521 × F2)	5.521
	*COD* = 85.338 + (1.444 × F1) − (0.295 × F2)	0.991
	*FM* = 83.255 + (1.412 × F1) + (2.187 × F2)	2.710
	*TSS* = 81.022 − (4.507 × F1) + (2.407 × F2) − (2.518 × F3)	5.172
	*COD* = 85.338 + (1.439 × F1) − (0.287 × F2) + (0.178 × F3)	0.957
	*FM* = 83.255 + (1.319 × F1) + (2.333 × F2) + (3.225 × F3)	1.021
	*TSS* = 81.022 − (4.526 × F1) + (2.425 × F2) − (2.487 × F3) + (0.771 × F4)	5.159
	*COD* = 85.338 + (1.444 × F1) − (0.293 × F2) + (0.169 × F3) − (0.231 × F4)	0.917
	*FM* = 83.255 + (1.326 × F1) + (2.326 × F2) + (3.214 × F3) − (0.286 × F4)	0.978

A MAPE of less than 5% is considered an indication that the prediction is acceptably accurate. A MAPE larger than 10% but less than 25% suggests low but acceptable accuracy, and a MAPE greater than 25% shows very low accuracy, so low that the prediction is not acceptable in terms of its accuracy [54].

## Data Availability

Not applicable.
